# Untargeted Metabolomics for Integrative Taxonomy: Metabolomics, DNA Marker-Based Sequencing, and Phenotype Bioimaging

**DOI:** 10.3390/plants12040881

**Published:** 2023-02-15

**Authors:** Kristian Peters, Kaitlyn L. Blatt-Janmaat, Natalia Tkach, Nicole M. van Dam, Steffen Neumann

**Affiliations:** 1 German Centre for Integrative Biodiversity Research (iDiv) Halle-Jena-Leipzig, Puschstrasse 4, 04103 Leipzig, Germany; 2 Institute of Biology/Geobotany and Botanical Garden, Martin Luther University Halle-Wittenberg, Am Kirchtor 1, 06108 Halle, Germany; 3 Bioinformatics and Scientific Data, Leibniz Institute of Plant Biochemistry, Weinberg 3, 06120 Halle, Germany; 4 Department of Chemistry, University of New Brunswick, Fredericton, NB E3B 5A3, Canada; 5 Institute of Biodiversity, Friedrich Schiller University Jena, Dornburgerstraße 159, 07743 Jena, Germany; 6 Plants Biotic Interactions, Leibniz Institute of Vegetable and Ornamental Crops (IGZ), Theodor-Echtermeyer-Weg 1, 14979 Großbeeren, Germany

**Keywords:** biodiversity, bryophytes, liverworts, chemophenetics, chemotaxonomy, ecological metabolomics, phylogenetics, bioimaging, phenotypes, sequencing, FAIR data

## Abstract

Integrative taxonomy is a fundamental part of biodiversity and combines traditional morphology with additional methods such as DNA sequencing or biochemistry. Here, we aim to establish untargeted metabolomics for use in chemotaxonomy. We used three thallose liverwort species *Riccia glauca*, *R. sorocarpa*, and *R. warnstorfii* (order Marchantiales, Ricciaceae) with *Lunularia cruciata* (order Marchantiales, Lunulariacea) as an outgroup. Liquid chromatography high-resolution mass-spectrometry (UPLC/ESI-QTOF-MS) with data-dependent acquisition (DDA-MS) were integrated with DNA marker-based sequencing of the *trn*L-*trn*F region and high-resolution bioimaging. Our untargeted chemotaxonomy methodology enables us to distinguish taxa based on chemophenetic markers at different levels of complexity: (1) molecules, (2) compound classes, (3) compound superclasses, and (4) molecular descriptors. For the investigated *Riccia* species, we identified 71 chemophenetic markers at the molecular level, a characteristic composition in 21 compound classes, and 21 molecular descriptors largely indicating electron state, presence of chemical motifs, and hydrogen bonds. Our untargeted approach revealed many chemophenetic markers at different complexity levels that can provide more mechanistic insight into phylogenetic delimitation of species within a clade than genetic-based methods coupled with traditional morphology-based information. However, analytical and bioinformatics analysis methods still need to be better integrated to link the chemophenetic information at multiple scales.

## 1. Introduction

Taxonomy is a fundamental part of biodiversity research that seeks to characterize, classify, and name biological species [1]. Integrative taxonomy combines traditional morphology-based taxonomy with additional methods such as DNA sequencing or the selection of chemophenetic markers using biochemistry [1]. While DNA sequencing has been applied to a wide range of species [2], certain groups such as bryophytic liverworts, including the herein investigated species of the genus *Riccia*, have been found to be very challenging to sequence, predominantly due a high abundance of glycosides, polyphenols, flavonoids, tannins, fatty acids, and other specialized metabolites that coprecipitate with some common DNA extraction procedures [3,4], or the presence of many DNAses [3]. Thus, for these challenging taxonomic groups, alternative methods are of high interest.

The liverwort genus *Riccia* L. consists of more than 200 species worldwide. A high degree of intrageneric variation has been observed in morphological, cytological, life history, and ecological characters [5,6]. As a result, taxonomic classification of the entire group has been ambiguous and based on morphological and anatomical characters such stature, habitus, spore size, and spore ornamentation. In addition, convergent evolution of these morphological characters has been observed [6]. Previous efforts to construct a molecular phylogeny of *Riccia* were based on the plastid *rbc*L and *trn*L-*trn*F regions, the *psb*A and *rps*4 genes, and the nuclear ribosomal gene that is included at the end of the 5.8 S subunit and ITS 2 for sequencing. These analyses identified the genus *Riccia* and various subgroups as monophyletic. Hence, there is currently no conclusive synthetic phylogeny available for the genus *Riccia* [5,6,7,8,9].

Seeing the difficulties of taxonomic classification of the genus *Riccia*, here, we investigate untargeted chemotaxonomy as a method in addition to traditional morphological and genetic methods. Chemotaxonomy involves the classification of biological species and reconstruction of their phylogenetic relationships using chemophenetic marker molecules [10,11,12,13]. It is based on the assumption that most morphologically defined species have a constant core metabolome that defines their individual responses irrespective of their geographic origin or ecology [14,15]. It also assumes that robust morphological properties correspond to chemical differences [10,14]. Chemotaxonomy has been applied successfully to vascular plants such as the genus *Doronicum* L. [16] and cryptogams such as lichens for nearly 50 years [17,18], usually focusing on specific compound classes. However, chemotaxonomy has not often been performed on bryophytes [11,19,20,21,22,23], as the natural product chemistry of bryophytes is still less explored than in other groups of plants [24]. This is due to the comparably large number of “unknown unknowns” (molecules for which neither the structure nor the identity is known) [25,26] and due to the fact that phylogenetically analogously important chemical classes such as phenols in flowers of vascular plants have not been identified yet in bryophytes [16].

Recent analytical and computational developments in mass spectrometry (MS) allow for the capture of nearly all low-molecular semi-polar molecules in biological species [27]. Untargeted LC/MS-MS techniques including Data Dependent Acquisition (DDA) coupled to *in silico* fragmentation tools like MetFrag [28] and machine-learning tools like SIRIUS [29] now allow for the acquisition, computational annotation, and classification of the majority of acquired molecules. This greatly facilitates the identification of biomarkers and compound classes that distinctly describe species [27,30] and provides insights into the rate of chemical evolution and diversification [31], and thus may resolve the phylogeny of difficult taxonomical groups and cryptic species [13]. Therefore, untargeted metabolomics can contribute to a more ecological representation of plant systematics and the classification of certain taxa [13,16]. For instance, Asakawa *et al.* [32] report chemotaxonomically relevant acetylenic fatty acids to be very abundant in *Riccia* species that rarely occur in other Marchantiophyta. Flavonoids such as apigenin and luteolin glucuronides have also been reported to be restricted within bryophytes to the family Ricciaceae [33,34], whereas Riccionidin A and B have also been found in the liverworts *Riccia duplex*, *Marchantia polymorpha*, and *Scapania undulata* [35].

In this paper, we (1) demonstrate the applicability of untargeted metabolomics techniques in the chemotaxonomy of understudied species groups. To this end, we chose *Riccia glauca* L., *R. sorocarpa* Bisch., and *R. warnstorfii* Limpr. ex Warnst. (order Marchantiales, Ricciaceae) and *Lunularia cruciata* (L.) Dumort. ex Lindb. (order Marchantiales, Lunulariacea) as an outgroup species [36]. These thallose liverworts are non-model species for which only a few discernable morphological characters are available for taxonomical classification [5,6,7]. Overall, the taxonomic status and phylogenetic relationships of these species are still unresolved, impeding the interpretation of relationships between their ecology and evolution [8]. We also (2) demonstrate the power of our approach for integrative taxonomy. Thus, we present minimum reference data integrating the three domains: (a) chemotaxonomy, for the estimation of molecular chemophenetic markers using untargeted metabolomics (LC/MS-MS); (b) bioimaging, for the assessment of phenotypes and to allow for an estimation of morphological, anatomical, and phenotypic characters; and (c) DNA sequencing, for the determination of the phylogenetic relationship, which we treat as ground-truth information.

## 2. Results

### 2.1. Phenotypic Analysis (Bioimaging)

**Figure 1:**
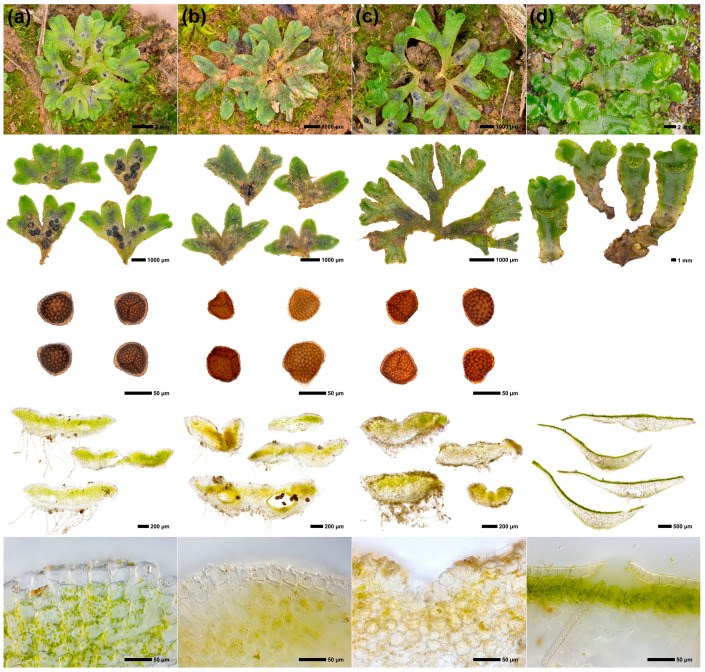
Overview of images showing main phenotypic characters of the investigated species. (**a**) *Riccia glauca*, (**b**) *R. sorocarpa*, (**c**) *R. warnstorfii*, (**d**) *Lunularia cruciata* outgroup. Phenotypic characters from top to bottom: growing stature, habitus of the ventral side of the thalli, spores (not obtained from *L. cruciata*), transverse section of the thallus, transverse section of the epidermis.

The bioimaging dataset consisted of a total of 15,615 raw images, 276 segmented images, and 40 fully processed images. Figure 1 shows an overview of the images of the main phenotypic characters.

**Figure 2:**
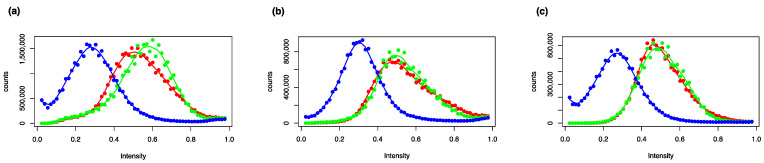
Spectra of habitus images of the thalli of *Riccia* species. The spectra are showing the histograms of the intensities of the red, green, and blue channels of the images. (**a**) *R. glauca*, (**b**) *R. sorocarpa*, (**c**) *R. warnstorfii*.

To demonstrate in principle how molecular traits can be linked to the phenotype, spectra of images of the statures (Figure 1, second row from the top) were determined for the *Riccia* species (Figure 2). Here, the thalli of the different species show different coloration (especially in the blue spectral components) (Figure 2).

**Figure 3:**
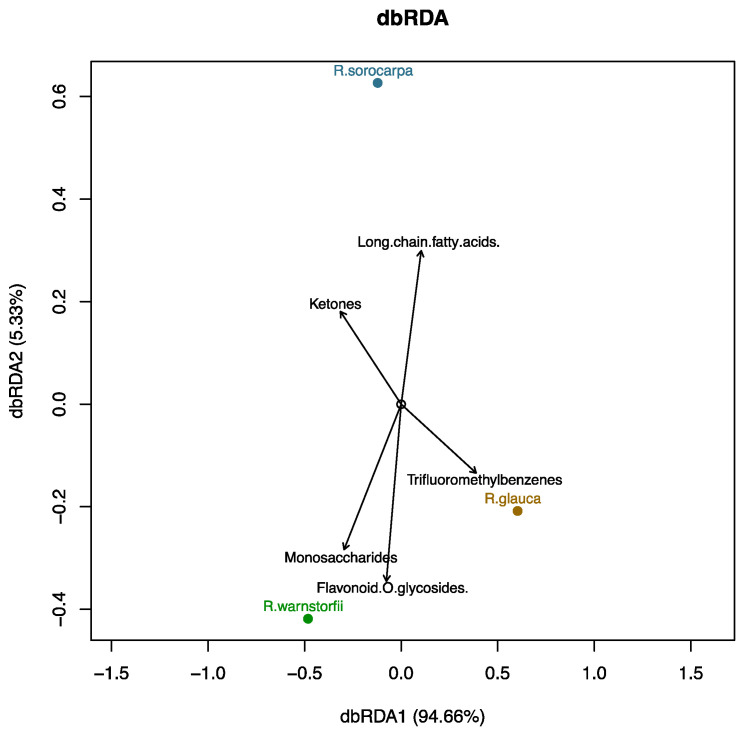
Ordination of spectral components in stature images of the three *Riccia* species and compound classes.

An ordination using distance-based redundancy analysis (dbRDA) was performed to obtain the molecular compound classes that correlate with the spectral components (Figure 3). The coloration of *R. glauca* was largely characterized by molecules of the class trifluoromethylbenzenes, whereas the other two species were characterized by specific high or low abundances in monosaccharides, specific flavonoid-glycosides, and long-chain fatty acids (Figure 3).

### 2.2. Chemotaxonomic Analysis Characterizing the Riccia Species Infragenerically

Metabolite profiles of the *Riccia* species were investigated with three biological replications for each species using untargeted high-resolution mass-spectrometry. A total of 6010 and 3671 metabolite features were successfully quantified in positive ion mode and negative ion mode, respectively. As metabolite features include redundant information on adducts, isotopes and in-source fragments, the profiles were subjected to a second stage of analytical fragmentation (data-dependent acquisition, or DDA-MS) which resulted in 442 high-quality MS2 fragment spectra in positive and negative ion modes for peaks detected in at least 70% of samples.

**Figure 4:**
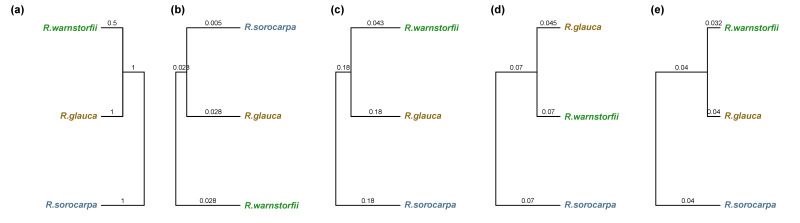
Chemotaxonomic trees characterizing the *Riccia* species infragenerically using chemophenetic markers at different levels. The numbers on the branches indicate edge lengths. Values in brackets indicate the results from the Mantel statistic *M* and the cophenetic correlation *c* comparing the chemotaxonomic trees with the phylogenetic tree. (**a**) Phylogenetic tree obtained using plastid DNA sequences of the *trn*L–*trn*F region, (**b**) tree obtained from the abundances of molecules (M = 0.624, c = 0.5), (**c**) tree obtained from the most specific compound classes (M = 0.634, c = 0.5), (**d**) tree obtained from molecules classified at the superclass level (M = 0.434, c = 0.5), (**e**) tree obtained from molecular descriptors (M = 0.688, c = 0.5).

**Figure 5:**
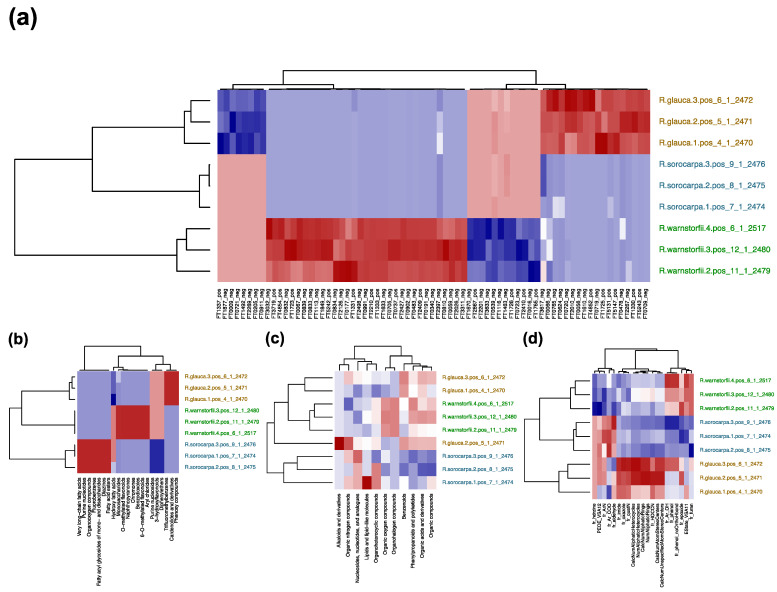
Heatmaps summarizing the results from variable selection of the chemophenetic markers characterizing the *Riccia* species infragenerically using PLS-DA at different levels. A red color indicates an overrepresentation and a blue color an underrepresentation of the variable. Values in brackets indicate the R-squared and the accuracy of the entire classification model. High-resolution, interactive, and zoomable plots are available in Zenodo [37]. Names of identified compounds are available in the Supplement. (**a**) Table of the abundances of molecules (R^2^ = 0.48, A = 0.889), (**b**) table of molecules classified in the most specific compound classes (R^2^ = 1.0, A = 1.0), (**c**) table of molecules classified in the superclass level (R^2^ = 0.871, A = 0.889), (**d**) table of molecular descriptors (R^2^ = 0.387, A = 0.778).

To select chemophenetic markers that characterize the three *Riccia* species at different levels, a metabolite feature table including the abundances of the MS1 precursors in positive and negative ion modes was used to obtain markers at the molecular level. The MS2 fragmentation data was used to identify markers using SIRIUS and to classify spectra at the compound class level using CANOPUS. Molecular descriptors were calculated for the annotated spectra and a descriptor table generated by performing a matrix operation with the feature table constrained for the annotated spectra. Chemotaxonomic trees were generated to compare chemotaxonomic results at different levels (Figure 4b–e) with the phylogenetic information obtained from DNA sequencing (Figure 4a). Significant chemophenetic markers were then selected using PLS-DA and visualized using heatmaps (Figure 5). Table S1 lists the selected chemophenetic molecules representative of the *Riccia* species at the molecular level.

The MS–MS fragment spectra in the infrageneric *Riccia* group were also checked for known compounds from the libraries MassBank [38], LOTUS [39], and KNApSAcK [40]. Table S2 summarizes these results.

### 2.3. Chemotaxonomic Analysis Characterizing the Riccia Species at the Genus Level from the Outgroup

**Figure 6:**
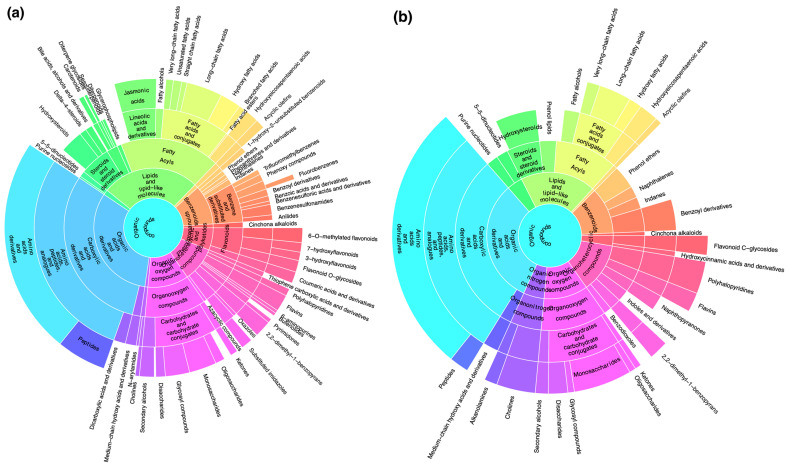
Sunburst plot showing the distribution of compound classes in (**a**) all *Riccia* species tested and (**b**) the outgroup species *Lunularia cruciata*. Interactive and zoomable sunburst plots are available in Zenodo [37].

Metabolite profiles were investigated as above, resulting in a total of 7340 metabolite features that were successfully quantified in positive ion mode and 4322 features in negative ion mode. Performing DDA-MS resulted in 682 high-quality MS–MS fragment spectra in positive and negative ion modes. Classification was performed using CANOPUS and resulted in a total of 103 annotated compound classes. The occurrences of the compound classes were counted and sunburst plots were generated for the *Riccia* species and the outgroup species *Lunularia cruciata* (Figure 6). The greatest differences were found in the compound classes of amino acids and derivatives, fatty acyls, glycosyl compounds, and benzenoids (Figure 6).

**Figure 7:**
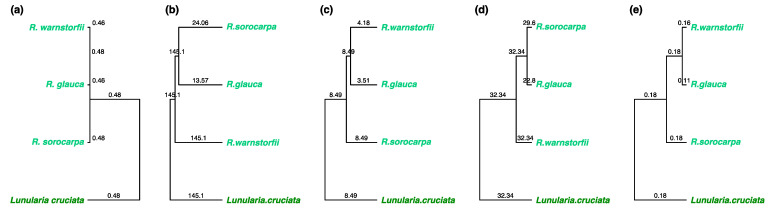
Chemotaxonomic trees of the *Riccia* species and the outgroup species *L. cruciata*; chemophenetic markers at different levels. The numbers on the branches indicate edge lengths. Values in brackets indicate the results from the Mantel statistic *M* and the cophenetic correlation *c* comparing the chemotaxonomic trees with the phylogenetic tree. (**a**) Phylogenetic tree obtained using the plastid DNA marker *trn*L–*trn*F, (**b**) tree obtained from the abundances of molecules (M = 0.38, c = 0.056), (**c**) tree obtained from the most specific compound classes (M = 0.12, c = 0.371), (**d**) tree obtained from molecules classified at the superclass level (M = 0.013, c = 0.209), (**e**) tree obtained from molecular descriptors (M = 0.215, c = 0.411).

**Figure 8:**
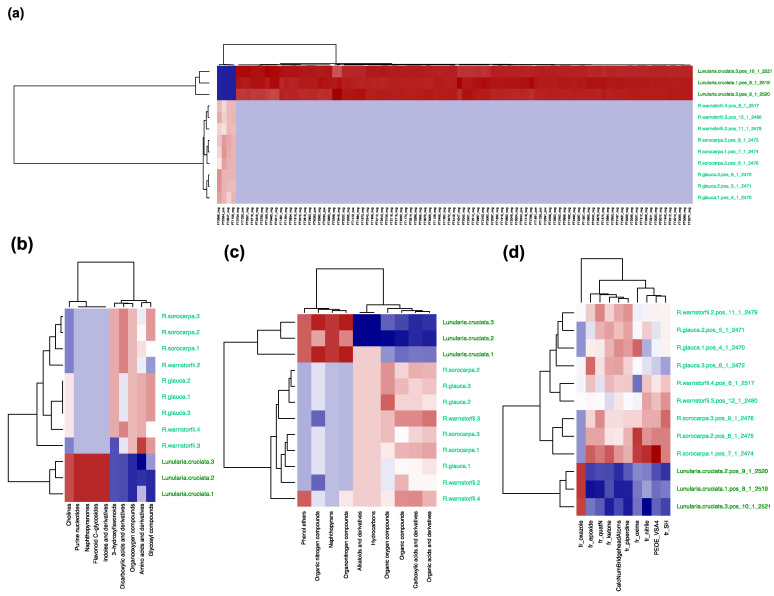
Heatmaps summarizing the results from variable selection of the chemophenetic markers comprising *Riccia* and the outgroup *L. cruciata* using PLS-DA at different levels. A red color indicates an overrepresentation and a blue color an underrepresentation of the variable. Values in brackets indicate the R-squared and the accuracy of the entire classification model. High-resolution, interactive, and zoomable plots are available in Zenodo [37]. Names of identified compounds are available in the Supplement. (**a**) Table of the abundances of molecules (R^2^ = 1.0, A = 1.0), (**b**) table of molecules classified in the most specific compound classes (R^2^ = 1.0, A = 1.0), (**c**) table of molecules classified in the superclass level (R^2^ = 1.0, A = 1.0), (**d**) table of molecular descriptors (R^2^ = 1.0, A = 1.0).

To select chemophenetic markers that characterize the three *Riccia* species at the genus level from the outgroup (represented by *Lunularia cruciata*), a metabolite feature table, computational classification tables, and molecular descriptors were determined as above. Chemotaxonomic trees were generated to compare results at different levels (Figure 7b–e) with the phylogenetic information obtained from DNA sequencing (Figure 7a). Significant chemophenetic markers were then selected using PLS-DA and visualized using heatmaps (Figure 8). Table S3 lists the selected chemophenetic markers separating the *Riccia* species from the outgroup at the molecular level.

### 2.4. DNA Sequence Analysis

**Figure 9:**
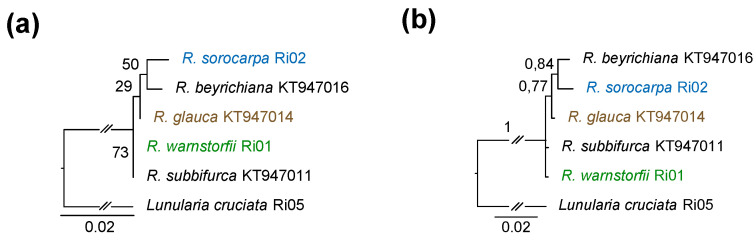
Phylogenetic tree based on plastid DNA *trn*L–*trn*F sequences of *Riccia* species and the outgroup *L. cruciata*. (**a**) Maximum likelihood phylogram, (**b**) Bayesian inferences phylogram. ML bootstrap support values and Bayesian posterior probabilities are indicated on the branches.

The plastid *trn*L–*trn*F sequence DNA dataset for six taxa included 550 aligned positions and contained new sequences of *Riccia sorocarpa* and *R. warnstorfii*, and *Lunularia cruciata* as an outgroup. The topology of the trees inferred by ML and BI analyses were largely identical, although their statistical supports of the ML tree were lower than of BI tree (Figure 9). Thus, the phylogenetic position of *R. glauca* could not be determined of the ML tree, whereas *R. warnstorfii* and *S. subbifurca* are sister taxa (Figure 9a). On the BI tree, *R. glauca* is sister to *R. beyrichiana and R. sorocarpa*, whereas the phylogenetic positions of *R. warnstorfii* and *R. subbifurca* are unresolved (Figure 9b).

## 3. Discussion

In this section, we discuss the three domains from which we integrated data and discuss novel insights from and the applicability of our untargeted chemotaxonomy approach for integrative taxonomy.

### 3.1. DNA Sequence Data

We performed DNA sequencing of the *trn*L–*trn*F plastid region of the three *Riccia* species and the outgroup species *L. cruciata* to obtain the phylogenetic relationships, which we treated as ground-truth information (the expected result) and compared it to the chemotaxonomy information at various levels. Prior to this study, sequencing data were not available for *R. warnstorfii*.

### 3.2. Bioimaging Data

Reference bioimaging data were generated from raw microscopic images and linked to technical and expressive metadata using standardized semantics [41,42,43,44]. When extracting phenotypic traits from bioimaging data, it is possible to estimate both quantitative traits (i.e., leaf and stem area, length, width of leaves, stems and plants, specific leaf area, specific stem density) and qualitative traits (i.e., growth stature, vegetative propagule, or leaf shape and type) by combining elemental analysis with machine-learning-driven image analysis and computer vision [45,46]. Recently, it has been shown that plant biomass accumulation can be predicted from image-derived parameters alone [47], making bioimaging analysis an emerging and powerful tool for various applications in ecology [48,49].

Here, we demonstrate how to investigate spectral components of stature images to obtain differences in the coloration of the thalli of the different species and how to relate this information to the chemical components found in the tested *Riccia* species. Under this exemplary framework, phenotypic and chemotaxonomic data could be integrated.

### 3.3. Chemotaxonomic Data

Over the past 10 years, tremendous progress has been made in the technology of (untargeted) metabolomics. Using mass spectrometry, it is now possible to measure and annotate nearly all low-molecular-weight (typically <1000 Da) semi-polar compounds in organisms at once without targeting specific compounds, covering a wide range of research questions [29,50,51]. Here, we used untargeted liquid chromatography high-resolution mass spectrometry (UPLC/ESI-QTOF-MS) with data-dependent acquisition (DDA-MS) and the computational annotation tool SIRIUS [29] to annotate and classify molecules, including metabolic compounds and related metabolite families [52]. Moreover, we also determined molecular descriptors for annotated compounds and discuss their role in characterizing the individual metabolic responses of species. Prior to this study, no metabolite profiles were available for any *Riccia* species. Data have been deposited to MetaboLights and are available as MTBLS4668.

In order to ensure a high level of quality, we subjected the data to extensive quality control (QC) to ensure that data were recorded and annotated correctly. This was accomplished by recording properties using biological replicates and by creating expressive metadata throughout the entire data-processing procedure. Metabolomics instrument performance and detection of batch effects in the metabolomics data was realized following an established QC protocol [53]. In the MetaboLights repository (study identifier MTBLS4668) [54], we provided blank samples at the beginning and the end of each chromatographic batch run to ensure that no significant shifts in mass-to-charge (m/z) and intensities had occurred during the run. We further provided samples with standard compounds (coumarins, MeOH, methanol) to validate known ionization properties to detect shifts and other effects in retention times and m/z. The QC pipeline of our data allows for re-analysis of standardized data in the context of large-scale chemotaxonomy studies [55].

### 3.4. Novel Insights from Untargeted Chemotaxonomy

Our principal study revealed large infrageneric molecular differences in the investigated *Riccia* species. This resulted in many chemophenetic markers that can potentially be used in chemotaxonomy. The *Riccia* species were collected from the same field site and, despite variations in environmental conditions likely being low, the metabolite profiles of *Riccia* were more dissimilar to those of *L. cruciata*, suggesting that *Riccia* taxa have a slightly more divergent metabolism. These results are in line with earlier reports for the genus [6]. This fact makes chemophenetic analyses very interesting for devising phylogenetic relationships within the genus *Riccia*. The high level of metabolic divergence also supports the view that liverworts in general are interacting predominantly at the metabolic level [56,57] through cryptic traits that do not manifest necessarily in the phenotype [31]. Analyzing the composition of compound classes in the different *Riccia* taxa at the level of subclasses [58] revealed similarly large infrageneric differences. This suggests that molecular differences can be generalized and that taxa have evolved characteristic strategies that mirror their phylogenetic status.

Phytochemical investigation and compound classification confirmed the presence of many acetylenic fatty acids (as shown in Figure 3) previously reported to be very characteristic for *Riccia* taxa and that rarely occur in other Marchantiophyta [32]. We also confirmed the presence of flavonoids such as apigenin and luteolin glucuronides (listed in Table S2) that have previously been reported to be restricted within bryophytes to the family of Ricciaceae [33,34]. Devising chemophenetic markers also offered us insight into rates of chemical evolution and diversification [31]. As early land plants, *Riccia* liverworts may have evolved unique glucuronide compounds serving as protection against excessive UV sunlight as has been shown for some glucuronides in *Marchantia polymorpha* [59]. Further, we could not detect Riccionidin A and B, which have previously been found in the liverworts *Riccia duplex*, *Marchantia polymorpha*, and *Scapania undulata* [35], suggesting that Riccionidins are either restricted to a few specialized taxa or only occur in low abundance in the species, which would not be detectable by our instrumental data-dependent acquisition setup. We also found many unique flavonoid glycosides and hydroxylated flavonoids in the *Riccia* profiles, which is in contrast to *Marchantia* spp. and *Lunularia* spp., which contain many unique stilbenes and (neo)lignans such as bisbibenzyls [27].

The annotation of untargeted LC/MS-MS data is still a complex task, as the natural product chemistry of bryophytes is not well known, and as a result, spectral libraries such as MassBank, GNPS, WeizMass, or Lotus only contain a few reference structures [24,38,60,61]. In order to unequivocally identify compounds, either authentic standards or additional elaborate analytical methods such as NMR are necessary [27]. However, in order to devise chemophenetic markers, we find that an annotation at the class level is sufficient to characterize the distinct *Riccia* taxa.

Here, we found a large infrageneric chemical diversity in the tested *Riccia* species. Although our analytical extraction method and the data-dependent acquisition were optimized to acquire plant metabolites, endophytic fungi may have contributed specialized metabolites to the overall phytochemical profiles [6,62], similarly by exogeneous mycorrhizal fungi [63,64]. We also cannot rule out secondary colonization by microbials, as the *Riccia* thalli were mature, and upon spore development, thalli release spores by becoming cavernous. Our experimental setup minimized contamination from exogenous and epiphytic species such as rhizoids, and any dirt and soil residues were remove from the thalli. The diversity of the chemical profiles may also be influenced by life stage, as *Riccia* samples were mature, containing spores.

Our principal investigation revealed that chemophenetic markers can be interpreted at different abstraction levels, providing different resolutions. Using untargeted metabolomics, we found that analyses at the more abstracted compound class and superclass levels [58] still provide a meaningful taxonomic interpretation [55]. However, care needs to be taken, as with every abstraction, variance is harmonized and yet may lead to the biased interpretation of overrepresented signals.

Overall, the investigated taxa displayed a high dissimilarity in their profiles. The PLS-DA model was able to differentiate the taxa at the molecule and subclass level with near-perfect separation. Evaluating the chemical composition at these two levels can thus support detailed insight into phylogenetic parentage of these taxa and be considered a viable alternative when genetic or morphological methods are inconclusive.

### 3.5. Applicability of Untargeted Chemotaxonomy

Our untargeted metabolomics methodology allowed us to distinguish taxa based on chemophenetic markers at different levels of complexity: (1) molecules, (2) compound classes, (3) compound superclasses, and (4) molecular descriptors. We aligned the results of the clade *Riccia* to the outgroup species *Lunularia cruciata* and compared the results at different levels with the reference information obtained from DNA sequencing. Our methodology is in contrast to many other chemotaxonomic studies that usually focus on only a few predominant classes, such as phenols [16,18]. In summary, we found large infrageneric differences in the tested *Riccia* species that were the result of distinctly produced molecules and marked differences in the composition of numerous compound classes. In conclusion, our data allowed us to devise chemophenetic markers using untargeted metabolomics at the molecular level based on presence–absence or based on abundances in the order of magnitude. Biomarker molecules are characterized by a specific mass-to-charge ratio that corresponds to the mass of the molecule, retention time, which is specific to the mass spectrometry, and the abundance, which corresponds to the ionization within the chromatographic column [65]. Once biomarkers have been determined, they can also be detected using FT-IR or thin-layer chromatography [20,21,66]. Moreover, we introduced compound classification to chemotaxonomy that allowed us to relate the composition and constitution of compound classes to biological taxa. While in the research field of eco-metabolomics, compound classification has become a powerful tool to generalize and simplify overly complex eco-molecular functioning [67], we conclude here that *in silico* compound classification is also applicable to resolve taxonomic relationships and may even be better suited than analytical approaches using fluorescence, spectrophotometers [68], or certain extraction procedures [69]. In this study, we investigated the metabolite profiles of three *Riccia* species growing at one location. In order to generalize findings and to devise chemophenetic biomarkers at different complexity levels, we recommend investigating species at several different locations to resolve the robustness of the chemophenetic markers. More research is clearly needed to assess and compare the resolution of these methods.

### 3.6. Integration of Untargeted Metabolomics into Integrative Taxonomy

In this study, we showed how chemotaxonomic data using untargeted metabolomics can be integrated into integrative taxonomy, which usually involves genetic and phenotypic data. Using untargeted metabolomics, we obtained chemophenetic markers at different levels of complexity: (1) molecules, (2) compound classes, (3) compound superclasses, and (4) molecular descriptors. We generated taxonomic trees from the data at the different levels and compared these trees with the reference information of taxonomic relationships of the species obtained from genetic markers. We found that chemotaxonomy can lead to more detailed information (more chemophenetic markers that distinctly characterize the different taxa) than the information from using genetic markers alone. However, the wealth of additional chemophenetic information needs to be carefully interpreted, as molecules can also represent individual responses of species to ecological factors, which may influence the taxonomic interpretation [70]. Thus, integrating the data for use in integrative taxonomy demands an evaluation of the metabolic state of the investigated taxa and an experiment design that minimizes any environmental or ecological influence. Lastly, by extracting spectral components of images of thallus phenotypes, we demonstrate in principle how differences in the coloration of the thalli relate to the molecular components found in the tested *Riccia* species.

## 4. Materials and Methods

### 4.1. Sample Collection and Processing

Samples of *Riccia glauca*, *R. sorocarpa*, and *R. warnstorfii* were collected by Uwe Schwarz from an arable stubble field near Aichtal Grötzingen in Baden-Württemberg, Germany on 09/13/2021 (geographic coordinates: 48.638275 N, 9.2534083 E, elevation: 376 m, precision: 10 m). *Lunularia cruciata* (L.) Dumort. ex Lindb. was additionally sampled near the lab site on 12/08/2021 at 51.494848 N, 11.942323 E and chosen as the outgroup species. The specimens were brought to the lab at IPB in sterile petri dishes, where plant material was isolated, washed under a light microscope to remove dirt and other residues, filled into Eppendorf tubes, and shock-frozen. Voucher specimens were stored in the herbarium Haussknecht Jena (voucher id’s: *R. glauca*: JE04010991, *R. sorocarpa*: JE04010990, *R. warnstorfii*: JE04010989, *L. cruciata*: JE04010993). For the metabolomics analysis, three biological replicates were used for each specimen. Table S4 gives an overview on samples and their use for the different types of analyses.

### 4.2. DNA Sequence Analysis

From the voucher specimens, small samples were taken for phylogenetic sequencing analyses (Table S4). DNA was extracted from herbarium specimens using 7–17 mg per sample. Total genomic DNA was extracted using the DNeasy Plant Mini Kit in accordance to the manufacturer’s protocol (Qiagen, Hilden, Germany). DNA concentration was checked with a NanoDrop spectrophotometer (2.2–6.6 ng/µL) and Invitrogen Qubit 3.0 fluorometer (0.73–4.92 ng/µL) (both ThermoFisher, Foster City, CA, USA). We tested four markers used in previous studies on *Riccia* and other liverworts: (1) the entire internal transcribed spacer region (ITS) of the nuclear ribosomal (nr) DNA (ITS1–5.8S rRNA gene–ITS2) of ca. 600 bp in length (primers ITS1 and ITS 4) [71]; (2) the end of the 5.8S subunit and ITS 2 (ca. 300 bp in length) (primers 5.8F and LS4-R) [7,72]; (3) the plastid *trn*K–*psb*A intergenic spacer and part of the *psb*A gene (primers trnK2F and 576F) [7,73]; (4) the plastid non-coding region of *trn*L–*trn*F, including the *trn*L(UAA) intron and the adjacent intergenic spacer between the *trn*L(UAA) 3′exon and *trn*F (GAA) gene (primers c and f) [7,73,74]. The following settings were used for the PCR reactions: 3 min at 94 °C, followed by 35 cycles of 30 s at 94 °C, 1 min at 50–53 °C, 1 min at 72 °C, and a final extension for 10 min at 72 °C. The sequencing was performed by LGC Genomics (Berlin, Germany). Although amplifications sometimes were successful, sequencing results were obtained only for the *trn*L–*trn*F marker for *R. sorocarpa*, *R. warnstorfii*, and *Lunularia cruciata*. Sampling was extended by the addition of three further *trn*L–*trn*F sequences taken from GenBank of *R. beyrichiana* (KT947016), *R. glauca* (KT947014), and R*. subbifurca* (KT947011) [8].

All new sequences were edited by eye in Sequencher 5.0 (Gene Codes Corporation, Ann Arbor, USA). The automatically performed alignment of six taxa was manually adjusted in Geneious 9.1.6 [75]. The phylogenetic reconstructions for the plastid region of *trn*L–*trn*F were conducted with Maximum Likelihood (ML) and Bayesian Inference (BI) methods. ML searches and bootstrap estimations of clade support were conducted with RAxML 8.2 [76] using RAxML BlackBox with default settings on the CIPRES Science Gateway [77]. On the same platform, the software MrBayes version 3.2.7a was used with the following parameters: rates = invgamma, ngen = 10,000,000, samplefreq = 1000 to estimate the posterior probabilities (PP) of the Bayesian analyses. The trees were visualized with FigTree 1.4.4 (https://github.com/rambaut/figtree; accessed on 12 February 2023). Sequencing data was deposited to Genbank and is available under the following accession numbers: *R. sorocarpa* OQ318168, *R. warnstorfii* OQ318167, *L. cruciata* OQ318169.

### 4.3. Phenotypic Analysis (Bioimaging)

Acquisition of images was based on the methods described in [78] and was only slightly modified for thallose liverwort species. In short, a Zeiss Axio Scope.A1 microscope was used for brightfield microscopy. For macroscopy and for preparing microscopy slides, a binocular microscope Zeiss Stemi 2000c was used. For macroscopic images, the Venus Optics Laowa 25 mm 2.5–5.0× ultra-macro for Canon EF was used. Digital images were acquired with a full-frame, high-resolution camera (Canon EOS RP, 26 megapixel).

To construct images with extended depth-of-field, images were recorded at focal planes at different z-layers. Raw images were pre-processed with Adobe Camera RAW and then exported to TIFF format while recording any image processing steps as metadata in Adobe XMP format. Multi-focus image fusion and image stitching were performed to improve the resolution of the final images Helicon Focus 8.1.1 (https://www.heliconsoft.com/heliconsoft-products/helicon-focus/; accessed on 12 February 2023) and Affinity Photo 1.10.5 (https://affinity.serif.com/en-us/photo/; accessed on 12 February 2023).

Images were manually segmented and interfering background removed using the flood select, brush selection, and freehand selection tools in the software Affinity Photo. Microscopic scales were then placed onto the segmented images using the approach described in [78].

Image features were estimated using the R package EBImage [79] by extracting the histograms of the red, green, and blue channels of the bioimages representing the visible spectra of the thalli of the different species. Distance-based ReDundancy Analyses (dbRDA) were performed using the *dbrda* function of the package vegan to investigate relationships of the image properties and the molecular traits [50]. Spectral values other than pure black (all RGB channels zero) and pure white (all RGB channels one) were extracted from the histogram models and used as traits in a dbRDA model. A Euclidean distance measure was used for the ordination. The dbRDA model with the largest explained variance was chosen using forward variable selection and the *ordistep* function. The goodness of fit statistic (squared correlation coefficient) was determined for the remaining variables by applying the *envfit* function on the dbRDA ordination model.

Raw camera and pre-processed imaging data were deposited to the BioImage Archive (BioStudies) [41] and are available under the identifier S-BIAD443 (https://www.ebi.ac.uk/biostudies/studies/S-BIAD443; accessed on 12 February 2023). Processed images and metadata were deposited to the Image Data Resource under accession number idr0137 [80,81].

### 4.4. Untargeted Metabolomics

#### 4.4.1. Metabolite Extraction and Untargeted Mass-Spectrometry

We followed extraction procedures for LC/MS originally developed for vascular plants by [82] and modified slightly for bryophytes [53]. This method has been shown to provide robust results for the compound classes we studied [83]. A detailed description of the protocol and methods can be found in [84]. In brief, frozen plants were homogenized in a ball mill at 25 Hz for 50 s and extracted with 1 mL of 80:20 MeOH:H_2_O. Samples were shaken at room temperature for 15 min at 1000 rpm, then sonicated for 15 min and shaken again for 15 min at 1000 rpm. Samples were centrifuged for 15 min at 13,000 rpm for 15 min; 750 µL of the supernatant was collected and concentrated in vacuo. Then, they were reconstituted to 10 mg fresh weight/100 µL with 80:20 MeOH:H2O and injected into a Bruker Elite HPLC equipped with a Nucleodur X18 Gravity-SB column (1.8 µm 100 × 2 Macherey Nagel, Dueren, Germany) and coupled to a Bruker TIMS-TOF (timsTOF Pro, Bruker, Bremen, Germany). Separate injections were performed for the positive and negative mode. Data-dependent acquisition (DDA-MS) mode was used with the instrument settings described in [84]. Due to different injection order, the second to fourth samples of *R. warnstorfii* were used for the analysis.

#### 4.4.2. Raw Data and MS1 Data Processing

Raw data converted into mzML format using msconvert [85] as well as derived data (SIRIUS project folders, RData) were deposited in MetaboLights under the study identifier MTBLS4668 [54]. Metadata were recorded in compliance with the minimum information guidelines for Metabolomics studies [86].

Data processing was performed in the statistical software environment R version 4.1.2 using the iESTIMATE framework (https://github.com/ipb-halle/iESTIMATE; accessed on 12 February 2023). Chromatographic peak detection was performed using the R package XCMS version 3.14.0 [87]. The following settings were used for the positive ion mode: CentWaveParam, ppm = 9.5, mzCenterFun = “mean”, peakwidth = c(4, 21), prefilter = c(2, 100), mzdiff = 0.0034, snthresh = 11, noise = 0, integrate = 1, firstBaselineCheck = TRUE, verboseColumns = FALSE, fitgauss = FALSE, roiList = list(), roiScales = numeric()); and for the negative ion mode: CentWaveParam, ppm = 9.5, mzCenterFun = “mean”, peakwidth = c(4, 36), prefilter = c(2, 170), mzdiff = 0.0045, snthresh = 11, noise = 0, integrate = 1, firstBaselineCheck = TRUE, verboseColumns = TRUE, fitgauss = FALSE, roiList = list(), roiScales = numeric(). Grouping of chromatographic peaks was performed before and after retention time correction with the following settings in both ion modes: PeakDensityParam, minFraction = 0.7, bw = 0.25. The retention time correction between the different profiles was performed with the following settings: PeakGroupsParam, minFraction = 0.7, smooth = “loess”, span = 0.2, family = “gaussian”. Only metabolite features with retention times less than 1020 s were considered for further analysis.

The MS1-level peak tables were created separately for positive and negative ion modes with the settings featureValues, method = “medret”, value = “into”. The peak tables were log-transformed, and missing values were imputed with zeros. Histograms and PCA diagnostic plots were generated to additionally evaluate the distribution of the data. MS2-level fragment spectra (MS/MS spectra) that were acquired by the Data-Dependent Acquisition mode (DDA-MS) were extracted from the profiles using the chromPeakSpectra, msLevel = 2 L, return.type = “Spectra” settings of XCMS. Spectra obtained from the same precursor ion were combined using the combineSpectra function from the R package Spectra using the following settings: FUN = combinePeaks, ppm = 35, peaks = “union”, minProp = 0.8, intensityFun = median, mzFun = median, backend = MsBackendDataFrame. This step was performed separately for positive and negative ion modes. The MS1-level peak tables were then filtered to include only peaks for which the DDA-MS had acquired MS/MS fragment spectra. The spectra were saved in MSP and MGF files for further data processing.

As standard variance and median values were within 10% deviations, the filtered MS1-level peak tables containing log-transformed abundances of peaks in positive and negative ion modes were joined and used for further statistical analyses. Presence/absence peak tables were also generated to contain whether a metabolite feature was detected in the profiles. Features with abundances less than 10^−8^ % of the median abundance were considered absent.

#### 4.4.3. Processing of MS/MS Data

Identification of MS/MS fragment spectra was carried out using the software SIRIUS version 5.6 [29]. The following settings were used for both ionizations: IsotopeSettings.filter = true, FormulaSearchDB, Timeout.secondsPerTree = 0, FormulaSettings.enforced = HCNOP, Timeout.secondsPerInstance = 0, UseHeuristic.mzToUseHeuristicOnly = 650, AlgorithmProfile = qtof, IsotopeMs2Settings = IGNORE, MS2MassDeviation.allowedMassDeviation = 10.0 ppm, NumberOfCandidatesPerIon = 1, UseHeuristic.mzToUseHeuristic = 300, FormulaSettings.detectable = B,Cl,Br,Se,S, NumberOfCandidates = 50, ZodiacNumberOfConsideredCandidatesAt300Mz = 10, ZodiacRunInTwoSteps = true, ZodiacEdgeFilterThresholds.minLocalConnections = 10, ZodiacEdgeFilterThresholds.thresholdFilter = 0.95, ZodiacEpochs.burnInPeriod = 2000, ZodiacEpochs.numberOfMarkovChains = 10, ZodiacNumberOfConsideredCandidatesAt800Mz = 50, ZodiacEpochs.iterations = 20,000, StructureSearchDB = ALL,BIO, FormulaResultThreshold = true, RecomputeResults = true, formula, zodiac, structure, canopus. For positive ion mode the settings were used: AdductSettings.detectable = [[M-H_4_O_2_ + H]+, [M + Na]+, [M + H]+, [M-H_2_O + H]+, [M + K]+, [M + H3N + H]+], AdductSettings.fallback = [[M + Na]+, [M + H]+, [M]+, [M + K]+]; and for negative mode: AdductSettings.detectable = [[M + Cl]−, [M-H_2_O-H]−, [M-H]−, [M + Br]−], AdductSettings.fallback = [[M + Cl]−, [M-H]−, [M]−, [M + Br]−].

Identification was accomplished automatically by selecting the highest-ranking candidate for each spectrum. If the software could provide a COSMIC score [30], the candidate with the highest-ranking COSMIC score was selected. The corresponding SMILES and the compound classes provided by the CANOPUS [88] were extracted and stored for each spectrum. In addition, biomarkers were manually identified, and the most likely library match for bryophytes or plants was manually curated.

The classification provided by CANOPUS for each MS/MS fragment spectrum was aggregated and stored in a separate classification table. Compound classes were analyzed at the ChemOnt level of subclasses and superclasses. The classes were aggregated and counted for each spectrum found in a sample and multiplied by the peak abundances of the corresponding MS1 precursors.

The SMILES provided by the SIRIUS software for the MS/MS fragment spectra were saved to a text file and molecular descriptors were calculated using RDKit and its Python module [89]. The RDKit results were saved in a csv file, which in turn was analyzed in R. A data table was constructed corresponding to the feature table by performing a matrix operation of both tables. This data table was used for performing statistical analyses (see below).

#### 4.4.4. Chemodiversity Analyses

To assess the overall chemical diversity, the richness was first determined representing the number of features, compounds, classes, or descriptors found in a sample, respectively. Second, the number of unique variables was determined that represents those variables that are present in one species but not the others. As a third diversity measure, the Shannon diversity index *H’* was determined according to [90]. Finally, the Pielou’s evenness *J* that describes the homogeneity of the distribution of the intensity or abundance of compounds present in a species was determined according to [90]. To assess significant differences among the groups, ANOVA with post-hoc Tukey honestly significant difference (HSD) test was calculated, and the R packages vegan, multcomp, and multtest were used.

To get an overview on the chemical diversity of compound classes and their diversity among or across species, sunburst plots were constructed. They were implemented as a custom function [91] comprised as stacked barplots from the inside out, starting with organic compounds in the center. The classes further to the outside represented the more specialized classes. The classes were arranged at different levels based on the CHEMONT ontology [58].

#### 4.4.5. Explorative and Unsupervised Multivariate Analyses

To discriminate species based on chemophenetic markers at different levels, principal components analysis (PCA) was performed using the prcomp function in R. In order to assess the influence of different study factors, variation partitioning was performed using the function varpart in the package vegan.

#### 4.4.6. Selection of Chemophenetic Molecular Features

Chemophenetic markers were selected at the levels of MS1 features (“feature list”), MS1 features constrained to the availability of MS2 spectra (“compound list”), at the compound class and superclass levels (“class list”, “superclass list”), and at the level of molecular descriptors (“descriptor list”). Variable selection was accomplished with the Partial Least Squares Discriminant Analysis (PLS-DA) using the caret package. A prediction model was trained using the train function from the caret package, and variable importance values were extracted from the model using the varImp function. Variables were selected (were considered significant) when their quantile threshold was above 0.995. In order to visualize significant relationships of the chemophenetic markers at the different levels, heatmaps were generated (using the gplots R package) from the selected variables.

To evaluate the performance of the fitted models, 10-fold cross-validation was performed (package mltest), and the Receiver Operating Characteristic (ROC) and PR (Precision and Recall) curves using the functions plot.roc and ci.se from the pROC package and the function pr.curve from the PRROC package were additionally constructed [92,93,94,95]. The R-squared of the fitted vs. the entire model and the area under curve (AUC) were calculated from the ROC, and the area under precision recall curve (AUC-PR) was determined from the PR curve.

#### 4.4.7. Construction of Taxonomic Trees

Taxonomic trees were constructed by first calculating a distance matrix on the feature tables using the Euclidean distance measure, and then, clustering was performed using the complete method. The following R packages were used: ape, pvclust, dendextend, phangorn, Hmisc, gplots.

#### 4.4.8. Deposition of Metabolomics Data

Raw metabolite profiles and the annotated feature tables were deposited in the MetaboLights repository (study identifier MTBLS4668) [54], along with QC samples consisting of blanks that were acquired at the beginning and at the end of each chromatographic batch run and samples containing standard compounds (coumarins, MeOH, methanol). Code to reproduce the results is available in GitHub (https://github.com/ipb-halle/iESTIMATE; accessed on 12 February 2023) [96].

## 5. Conclusions

Data on phenotypic, phylogenetic, and molecular traits of bryophytes are scarce but are needed to understand the individual responses of bryophytes with regard to characterizing, classifying, and naming species [1,97]. Integrating data that span multiple spatiotemporal scales, such as phenotypes, molecules, or DNA sequences is a key concept in integrative biodiversity research and will allow further linking of molecular processes to taxonomy and association of specific mechanistic characters of the species with their ecology and evolution [98]. In order to promote data re-use, we followed the FAIR principles, associated the datasets with rich metadata, and provide computational code to semi-automatically (re-)process the data [99,100]. Integrative taxonomy typically combines an assessment of phenotypes and DNA sequence markers to elucidate phylogenetic relationships of species [22,98]. In this study, we integrated untargeted metabolomics with DNA sequencing and phenotypic bioimages and show in principle how this integration allows for a more detailed taxonomic evaluation of the genus *Riccia*. We also showed how chemophenetic data allows for more realistic species circumscriptions [13]. The integrative data also allows investigation of the ecology and evolution of the species and can shed light on their origin and biogeographic history. Additionally, the integrative data will advance many related research areas such as functional ecology by investigating molecular traits [55], aiding global biodiversity synthesis efforts at various scales [101,102], and making connections between high-throughput biodiversity inventories to “classic” bryology, (digital) “collectomics” (“digitomics”), or data science [103]. The data may also be used in bioinformatics to train machine-learning models that may advance automated high-throughput analyses and pattern recognition [49].

## Data Availability

Raw camera and pre-processed imaging data were deposited to the BioImage Archive (BioStudies) and are available under the identifier S-BIAD443 (https://www.ebi.ac.uk/biostudies/studies/S-BIAD443). Processed images and metadata were deposited to the Image Data Resource under accession number idr0137 (https://doi.org/10.17867/10000185). Sequencing data were deposited to Genbank and are available under the following accession numbers: *R. sorocarpa* OQ318168, *R. warnstorfii* OQ318167, *L. cruciata* OQ318169. Raw metabolite profiles and the annotated feature tables were deposited to the MetaboLights repository under the study identifier MTBLS4668 (https://www.ebi.ac.uk/metabolights/MTBLS4668; accessed on 12 February 2023). Data analysis plots, images, and R code to reproduce the plots are available in Zenodo (https://doi.org/10.5281/zenodo.7638304). Source code is available on GitHub (https://doi.org/10.5281/zenodo.7615220).

## Supplementary Materials

The following supporting information can be downloaded at https://www.mdpi.com/article/10.3390/plants12040881/s1: Table S1: 71 chemophenetic marker molecules selected by PLS-DA characterizing the investigated *Riccia* species. Columns include the internal identifier used by the XCMS peak detection software, the mass-to-charge ratio of the precursor ion (m/z), retention time (RT) (s), compound name, the most specific compound class, the SMILES, and the level of annotation confidence (MSI level) according to [104]; Table S2: Compounds of interest. Table containing known compounds that were previously described in the literature to be characteristic for *Riccia* species; Table S3: Chemophenetic biomarker molecules selected by PLS-DA representative of each of the investigated *Riccia* species. Columns include the internal identifier used by the XCMS peak detection software, the mass-to-charge ratio of the precursor ion (m/z), retention time (RT) (s), compound name, the most specific compound class, the SMILES, and the level of annotation confidence (MSI level) according to [104]; Table S4: List of samples and their identification codes for use with the different types of analyses.

## References

[1] Schlick-Steiner B.C., Steiner F.M., Seifert B., Stauffer C., Christian E., Crozier R.H. (2010). Integrative Taxonomy: A Multisource Approach to Exploring Biodiversity. Annu. Rev. Entomol..

[2] One Thousand Plant Transcriptomes Initiative (2019). One thousand plant transcriptomes and the phylogenomics of green plants. Nature.

[3] Jordon-Thaden I.E., Chanderbali A.S., Gitzendanner M.A., Soltis D.E. (2015). Modified CTAB and TRIzol protocols improve RNA extraction from chemically complex Embryophyta. Appl. Plant Sci..

[4] Križman M., Jakše J., Baričevič D., Javornik B., Prošek M. (2006). Robust CTAB-activated charcoal protocol for plant DNA extraction. Acta Agric. Slov..

[5] Cargill D.C., Beckmann K., Seppelt R. (2021). Taxonomic revision of *Riccia* (Ricciaceae, Marchantiophyta) in the monsoon tropics of the Northern Territory, Australia. Aust. Syst. Bot..

[6] Wheeler J.A. (2000). Molecular Phylogenetic Reconstructions of the Marchantioid Liverwort Radiation. Bryologist.

[7] Cargill D.C., Neal W.C., Sharma I., Gueidan C. (2016). A preliminary molecular phylogeny of the genus *Riccia* L. (*Ricciaceae*) in Australia. Aust. Syst. Bot..

[8] Dirkse G.M., Losada-Lima A., Stech M. (2016). *Riccia boumanii* Dirkse, Losada & M.Stech *sp. nov*. (Ricciaceae, Marchantiophyta) in the Canary Islands, the first species of *Riccia* subgenus *Riccia* section *Pilifer* Volk outside South Africa. J. Bryol..

[9] Hinchliff C.E., Smith S.A., Allman J.F., Burleigh J.G., Chaudhary R., Coghill L.M., Crandall K.A., Deng J., Drew B.T., Gazis R. (2015). Synthesis of phylogeny and taxonomy into a comprehensive tree of life. Proc. Natl. Acad. Sci. USA.

[10] Fox H.M. (1946). Chemical Taxonomy. Nature.

[11] McClure J.W., Miller H.A. (1967). Moss chemotaxonomy. A survey for flavonoids and their taxonomicimplications. Nova Hedwig..

[12] Singh R. (2016). Chemotaxonomy: A Tool for Plant Classification. J. Med. Plants Stud..

[13] Zidorn C. (2019). Plant chemophenetics—A new term for plant chemosystematics/plant chemotaxonomy in the macro-molecular era. Phytochemistry.

[14] Brodo I.M. (1986). Interpreting Chemical Variation in Lichens for Systematic Purposes. Bryologist.

[15] Rogers R.W. (1989). Chemical variation and the species concept in lichenized ascomycetes. Bot. J. Linn. Soc..

[16] Willer J., Christensen E., Wahl A., Gemeinholzer B., Zidorn C. (2022). Phylogeny and chemophenetics of the newly described *Doronicum* × *longeflorens* and related *Doronicum* taxa (Senecioneae, Asteraceae). Biochem. Syst. Ecol..

[17] Culberson W.L. (1969). The use of chemistry in the systematics of the lichens. Taxon.

[18] Lumbsch H.T., Kranner I.C., Beckett R.P., Varma A.K. (2002). Analysis of Phenolic Products in Lichens for Identification and Taxonomy. Protocols in Lichenology.

[19] Figueiredo A.C., Sim-Sim M., Barroso J.G., Pedro L.G., Esquível M.G., Fontinha S., Luís L., Martins S., Lobo C., Stech M. (2009). Liverwort *Radula* species from Portugal: Chemotaxonomical evaluation of volatiles composition. Flavour Fragr. J..

[20] Hawrył A., Bogucka-Kocka A., Świeboda R., Hawrył M., Stebel A., Waksmundzka-Hajnos M. (2018). Thin-layer chromatography fingerprint and chemometric analysis of selected Bryophyta species with their cytotoxic activity. JPC J. Planar Chromatogr. Mod. TLC.

[21] Hu T., Jin W.-Y., Cheng C.-G. (2011). Classification of Five Kinds of Moss Plants with the Use of Fourier Transform Infrared Spectroscopy and Chemometrics. Spectroscopy.

[22] Lee G.E., Bechteler J., Pócs T., Schäfer-Verwimp A., Tang H.Y., Chia P.W. (2022). Integrative Taxonomy Reveals a New Species of the Genus *Lejeunea* (Marchantiophya: Lejeuneaceae) from Peninsular Malaysia. Plants.

[23] Ludwiczuk A., Raharivelomanana P., Pham A., Bianchini J.-P., Asakawa Y. (2014). Chemical variability of the Tahitian *Marchantia hexaptera* Reich. Phytochem. Lett..

[24] Asakawa Y., Ludwiczuk A. (2018). Chemical Constituents of Bryophytes: Structures and Biological Activity. J. Nat. Prod..

[25] da Silva R.R., Dorrestein P.C., Quinn R.A. (2015). Illuminating the dark matter in metabolomics. Proc. Natl. Acad. Sci. USA.

[26] Wishart D.S. (2009). Computational strategies for metabolite identification in metabolomics. Bioanalysis.

[27] Peters K., Balcke G., Kleinenkuhnen N., Treutler H., Neumann S. (2021). Untargeted In Silico Compound Classification—A Novel Metabolomics Method to Assess the Chemodiversity in Bryophytes. IJMS.

[28] Ruttkies C., Schymanski E.L., Wolf S., Hollender J., Neumann S. (2016). MetFrag relaunched: Incorporating strategies beyond in silico fragmentation. J. Cheminform..

[29] Dührkop K., Fleischauer M., Ludwig M., Aksenov A.A., Melnik A.V., Meusel M., Dorrestein P.C., Rousu J., Böcker S. (2019). SIRIUS 4: A rapid tool for turning tandem mass spectra into metabolite structure information. Nat. Methods.

[30] Hoffmann M.A., Nothias L.-F., Ludwig M., Fleischauer M., Gentry E.C., Witting M., Dorrestein P.C., Dührkop K., Böcker S. (2022). High-confidence structural annotation of metabolites absent from spectral libraries. Nat. Biotechnol..

[31] Sedio B.E. (2017). Recent breakthroughs in metabolomics promise to reveal the cryptic chemical traits that mediate plant community composition, character evolution and lineage diversification. New Phytol..

[32] Asakawa Y., Ludwiczuk A., Nagashima F., Asakawa Y., Ludwiczuk A., Nagashima F., Asakawa Y., Ludwiczuk A., Nagashima F. (2013). Fortschritte der Chemie organischer Naturstoffe = Progress in the chemistry of organic natural products. Chemical Constituents of Bryophytes: Bio- and Chemical Diversity, Biological Activity and Chemosystematics.

[33] Kohn G., Vandekerkhove O., Hartmann E., Beutelmann P. (1988). Acetylenic fatty acids in the *Ricciaceae* (Hepaticae). Phytochemistry.

[34] Markham K.R., J. Porter L. (1975). Evidence of biosynthetic simplicity in the flavonoid chemistry of the Ricciaceae. Phytochemistry.

[35] Kunz S., Burkhardt G., Becker H. (1993). Riccionidins a and b, anthocyanidins from the cell walls of the liverwort *Ricciocarpos natans*. Phytochemistry.

[36] Shaw A.J., Szovenyi P., Shaw B. (2011). Bryophyte diversity and evolution: Windows into the early evolution of land plants. Am. J. Bot..

[37] Peters K., Blatt-Janmaat K., Tkach N., Van Dam N.M., Neumann S. (2023). Investigating untargeted metabolomics for its use in integrative taxonomy—Linking metabolomics, DNA marker-based se-quencing and bioimaging of phenotypes. Zenodo.

[38] Horai H., Arita M., Kanaya S., Nihei Y., Ikeda T., Suwa K., Ojima Y., Tanaka K., Tanaka S., Aoshima K. (2010). MassBank: A public repository for sharing mass spectral data for life sciences. J. Mass Spectrom..

[39] Rutz A., Sorokina M., Galgonek J., Willighagen E., Gaudry A., Graham J.G., Stephan R., Page R., Vondrášek J., Steinbeck C. (2021). The LOTUS Initiative for Open Natural Products Research: Knowledge Management through Wikidata. BioRxiv.

[40] Nakamura Y., Mochamad Afendi F., Kawsar Parvin A., Ono N., Tanaka K., Hirai Morita A., Sato T., Sugiura T., Altaf-Ul-Amin M., Kanaya S. (2014). KNApSAcK Metabolite Activity Database for Retrieving the Relationships Between Metabolites and Biological Activities. Plant Cell Physiol..

[41] Ellenberg J., Swedlow J.R., Barlow M., Cook C.E., Sarkans U., Patwardhan A., Brazma A., Birney E. (2018). A call for public archives for biological image data. Nat. Methods.

[42] Löffler F., Wesp V., König-Ries B., Klan F. (2021). Dataset search in biodiversity research: Do metadata in data repositories reflect scholarly information needs?. PLoS ONE.

[43] Meijering E., Carpenter A.E., Peng H., Hamprecht F.A., Olivo-Marin J.-C. (2016). Imagining the future of bioimage analysis. Nat. Biotechnol..

[44] Samuel S., Taubert F., Walther D., König-Ries B., Bücker H.M. (2017). Towards Reproducibility of Microscopy Experiments. D-Lib Mag..

[45] Pérez-Harguindeguy N., Díaz S., Garnier E., Lavorel S., Poorter H., Jaureguiberry P., Bret-Harte M.S., Cornwell W.K., Craine J.M., Gurvich D.E. (2013). New handbook for standardised measurement of plant functional traits worldwide. Aust. J. Bot..

[46] Kommineni V.K., Tautenhahn S., Baddam P., Gaikwad J., Wieczorek B., Triki A., Kattge J. (2021). Comprehensive leaf size traits dataset for seven plant species from digitised herbarium specimen images covering more than two centuries. BDJ.

[47] Chen D., Shi R., Pape J.-M., Neumann K., Arend D., Graner A., Chen M., Klukas C. (2018). Predicting plant biomass accumulation from image-derived parameters. GigaScience.

[48] Hansen O.L.P., Svenning J., Olsen K., Dupont S., Garner B.H., Iosifidis A., Price B.W., Høye T.T. (2020). Species-level image classification with convolutional neural network enables insect identification from habitus images. Ecol. Evol..

[49] Høye T.T., Ärje J., Bjerge K., Hansen O.L.P., Iosifidis A., Leese F., Mann H.M.R., Meissner K., Melvad C., Raitoharju J. (2021). Deep learning and computer vision will transform entomology. Proc. Natl. Acad. Sci. USA.

[50] Peters K., Gorzolka K., Bruelheide H., Neumann S. (2018). Seasonal variation of secondary metabolites in nine different bryophytes. Ecol. Evol..

[51] Jarmusch S.A. (2021). Advancements in capturing and mining mass spectrometry data are transforming natural products research. Nat. Prod. Rep..

[52] Peters K., Worrich A., Weinhold A., Alka O., Balcke G., Birkemeyer C., Bruelheide H., Calf O., Dietz S., Dührkop K. (2018). Current Challenges in Plant Eco-Metabolomics. Int. J. Mol. Sci..

[53] Peters K., Gorzolka K., Bruelheide H., Neumann S. (2018). Computational workflow to study the seasonal variation of secondary metabolites in nine different bryophytes. Sci. Data.

[54] Haug K., Salek R.M., Conesa P., Hastings J., de Matos P., Rijnbeek M., Mahendraker T., Williams M., Neumann S., Rocca-Serra P. (2013). MetaboLights—An open-access general-purpose repository for metabolomics studies and associated meta-data. Nucleic Acids Res..

[55] Walker T.W.N., Alexander J.M., Allard P., Baines O., Baldy V., Bardgett R.D., Capdevila P., Coley P.D., David B., Defossez E. (2022). Functional Traits 2.0: The power of the metabolome for ecology. J. Ecol..

[56] Renner M.A. (2020). Opportunities and challenges presented by cryptic bryophyte species. Telopea.

[57] Shaw J. (2001). Biogeographic patterns and cryptic speciation in bryophytes: Cryptic speciation in bryophytes. J. Biogeogr..

[58] Djoumbou Feunang Y., Eisner R., Knox C., Chepelev L., Hastings J., Owen G., Fahy E., Steinbeck C., Subramanian S., Bolton E. (2016). ClassyFire: Automated chemical classification with a comprehensive, computable taxonomy. J. Cheminformatics.

[59] Soriano G., Del-Castillo-Alonso M.-Á., Monforte L., Tomás-Las-Heras R., Martínez-Abaigar J., Núñez-Olivera E. (2021). Developmental Stage Determines the Accumulation Pattern of UV-Absorbing Compounds in the Model Liverwort *Marchantia polymorpha* subsp. ruderalis under Controlled Conditions. Plants.

[60] Allard P.-M., Péresse T., Bisson J., Gindro K., Marcourt L., Pham V.C., Roussi F., Litaudon M., Wolfender J.-L. (2016). Integration of Molecular Networking and *In-Silico* MS/MS Fragmentation for Natural Products Dereplication. Anal. Chem..

[61] Shahaf N., Rogachev I., Heinig U., Meir S., Malitsky S., Battat M., Wyner H., Zheng S., Wehrens R., Aharoni A. (2016). The WEIZMASS spectral library for high-confidence metabolite identification. Nat. Commun..

[62] Stelmasiewicz M., Świątek Ł., Ludwiczuk A. (2021). Phytochemical Profile and Anticancer Potential of Endophytic Microorganisms from Liverwort Species, *Marchantia polymorpha* L.. Molecules.

[63] Wangikar H., Chavan S.J., Bankar P., Gavali P., Taware T. (2021). Analysis and fungal Isolation of some mosses, *Riccia discolor* and *Targionia hyophylla* from Baramati, district-Pune, Maharashtra, India. Int. J. Bot. Stud. 7.

[64] Wankhede Tb W.T. (2017). Mycorrhization in bryophyte riccia discolor lehm. et. lindenb. IJRBAT.

[65] Tautenhahn R., Bottcher C., Neumann S. (2008). Highly sensitive feature detection for high resolution LC/MS. BMC Bioinform..

[66] Klavina L. (2015). A study on bryophyte chemical composition–search for new applications. Agron. Res..

[67] Uthe H., van Dam N.M., Hervé M.R., Sorokina M., Peters K., Weinhold A. (2020). A practical guide to implementing metabolomics in plant ecology and biodiversity research. Advances in Botanical Research.

[68] Sabovljević M.S., Sabovljević A.D., Ikram N.K.K., Peramuna A., Bae H., Simonsen H.T. (2016). Bryophytes—An emerging source for herbal remedies and chemical production. Plant Genet. Resour..

[69] Khalkar K.M., Kadam V.B. (2021). Biochemical Evaluation of Some Liverworts Pigments and Phenolics. J. Drug Delivery Ther..

[70] van Dam N.M., van der Meijden E., Hall R.D. (2011). A Role for Metabolomics in Plant Ecology. Annual Plant Reviews Volume 43.

[71] White T.J., Bruns T., Lee S., Taylor J. (1990). Amplification And Direct Sequencing Of Fungal Ribosomal RNA Genes For Phylogenetics. PCR Protocols.

[72] Vanderpoorten A., Quandt D., Goffinet B. (2006). Utility of the Internal Transcribed Spacers of the 18S-5.8S-26S Nuclear Ribosomal DNA in Land Plant Systematics with Special Emphasis on Bryophytes. Plant Genome: Biodiversity and Evolution—Volume 2, Part B.

[73] Forrest L.L., Crandall-Stotler B.J. (2004). A phylogeny of the simple thalloid liverworts (Junger-manniopsida, subclass Metzgeriidae) as inferred from five chloroplast genes. Monogr. Syst. Bot. Mo. Bot. Gard..

[74] Taberlet P., Gielly L., Pautou G., Bouvet J. (1991). Universal primers for amplification of three non-coding regions of chloroplast DNA. Plant Mol. Biol..

[75] Kearse M., Moir R., Wilson A., Stones-Havas S., Cheung M., Sturrock S., Buxton S., Cooper A., Markowitz S., Duran C. (2012). Geneious Basic: An integrated and extendable desktop software platform for the organization and analysis of sequence data. Bioinformatics.

[76] Stamatakis A. (2014). RAxML version 8: A tool for phylogenetic analysis and post-analysis of large phylogenies. Bioinformatics.

[77] Miller M.A., Pfeiffer W., Schwartz T. (2010). Creating the CIPRES Science Gateway for inference of large phylogenetic trees. 2010 Gateway Computing Environments Workshop (GCE).

[78] Peters K., König-Ries B. (2022). Reference bioimaging to assess the phenotypic trait diversity of bryophytes within the family Scapaniaceae. Sci. Data.

[79] Pau G., Fuchs F., Sklyar O., Boutros M., Huber W. (2010). EBImage--an R package for image processing with applications to cellular phenotypes. Bioinformatics.

[80] Williams E., Moore J., Li S.W., Rustici G., Tarkowska A., Chessel A., Leo S., Antal B., Ferguson R.K., Sarkans U. (2017). Image Data Resource: A bioimage data integration and publication platform. Nat. Methods.

[81] Peters K. (2022). Data Integration in Biodiversity—A Principal Investigation on Three Liverwort Species of Riccia Integrating Metabolomics, Sequencing and Phenotypic Data for Use in Integrative Taxonomy.

[82] Böttcher C., Westphal L., Schmotz C., Prade E., Scheel D., Glawischnig E. (2009). The Multifunctional Enzyme CYP71B15 (PHYTOALEXIN DEFICIENT3) Converts Cysteine-Indole-3-Acetonitrile to Camalexin in the Indole-3-Acetonitrile Metabolic Network of Arabidopsis thaliana. Plant Cell. Online.

[83] Lu Y., Eiriksson F.F., Thorsteinsdóttir M., Simonsen H.T. (2021). Effects of extraction parameters on lipid profiling of mosses using UPLC-ESI-QTOF-MS and multivariate data analysis. Metabolomics.

[84] Blatt-Janmaat K.L., Neumann S., Schmidt F., Ziegler J., Peters K., Qu Y. (2022). Impact of in vitro hormone treatments on the bibenzyl production of Radula complanata. Botany.

[85] Chambers M.C., Maclean B., Burke R., Amodei D., Ruderman D.L., Neumann S., Gatto L., Fischer B., Pratt B., Egertson J. (2012). A cross-platform toolkit for mass spectrometry and proteomics. Nat. Biotechnol..

[86] Spicer R.A., Salek R., Steinbeck C. (2017). Compliance with minimum information guidelines in public metabolomics repositories. Sci. Data.

[87] Smith C.A., Want E.J., O’Maille G., Abagyan R., Siuzdak G. (2006). XCMS: Processing Mass Spectrometry Data for Metabolite Profiling Using Nonlinear Peak Alignment, Matching, and Identification. Anal. Chem..

[88] Dührkop K., Nothias L.-F., Fleischauer M., Reher R., Ludwig M., Hoffmann M.A., Petras D., Gerwick W.H., Rousu J., Dorrestein P.C. (2020). Systematic classification of unknown metabolites using high-resolution fragmentation mass spectra. Nat. Biotechnol..

[89] Bento A.P., Hersey A., Félix E., Landrum G., Gaulton A., Atkinson F., Bellis L.J., De Veij M., Leach A.R. (2020). An open source chemical structure curation pipeline using RDKit. J. Cheminform.

[90] Peters K., Poeschl Y., Blatt-Janmaat K.L., Uthe H., Murthy H.N. (2022). Ecometabolomics Studies of Bryophytes. Bioactive Compounds in Bryophytes and Pteridophytes.

[91] Peters K. (2019). Chemical Diversity and Classification of Secondary Metabolites in Nine Bryophyte Species. Metabolites.

[92] Fawcett T. (2006). An introduction to ROC analysis. Pattern Recognit. Lett..

[93] Grau J., Grosse I., Keilwagen J. (2015). PRROC: Computing and visualizing precision-recall and receiver operating characteristic curves in R. Bioinformatics.

[94] Robin X., Turck N., Hainard A., Tiberti N., Lisacek F., Sanchez J.-C., Müller M. (2011). pROC: An open-source package for R and S+ to analyze and compare ROC curves. BMC Bioinform..

[95] Tharwat A. (2020). Classification assessment methods. ACI.

[96] Peters K. (2023). iESTIMATE Computational Analysis Framework for Eco-Metabolomics Data Version 0.4. https://zenodo.org/record/7615220#.Y-xNKHZByUk.

[97] Stanton D.E., Coe K.K. (2021). 500 million years of charted territory: Functional ecological traits in bryophytes. BDE.

[98] Price S.A., Schmitz L. (2016). A promising future for integrative biodiversity research: An increased role of scale-dependency and functional biology. Phil. Trans. R. Soc. B.

[99] Goble C., Cohen-Boulakia S., Soiland-Reyes S., Garijo D., Gil Y., Crusoe M.R., Peters K., Schober D. (2020). FAIR Computational Workflows. Data Intell..

[100] Wilkinson M.D., Dumontier M., Aalbersberg I.J., Appleton G., Axton M., Baak A., Blomberg N., Boiten J.-W., da Silva Santos L.B., Bourne P.E. (2016). The FAIR Guiding Principles for scientific data management and stewardship. Sci. Data.

[101] Heberling J.M., Miller J.T., Noesgaard D., Weingart S.B., Schigel D. (2021). Data integration enables global biodiversity synthesis. Proc. Natl. Acad. Sci. USA.

[102] König C., Weigelt P., Schrader J., Taylor A., Kattge J., Kreft H. (2019). Biodiversity data integration—The significance of data resolution and domain. PLoS Biol..

[103] Arribas P., Andújar C., Bidartondo M.I., Bohmann K., Coissac É., Creer S., deWaard J.R., Elbrecht V., Ficetola G.F., Goberna M. (2021). Connecting high-throughput biodiversity inventories: Opportunities for a site-based genomic framework for global integration and synthesis. Mol. Ecol..

[104] Schymanski E.L., Jeon J., Gulde R., Fenner K., Ruff M., Singer H.P., Hollender J. (2014). Identifying Small Molecules via High Resolution Mass Spectrometry: Communicating Confidence. Environ. Sci. Technol..

