# Supplementary material for: Untargeted Metabolomics for Integrative Taxonomy: Metabolomics, DNA Marker-Based Sequencing, and Phenotype Bioimaging
Source: Plants (Basel). 2023 Feb 15;12(4):881. doi: 10.3390/plants12040881 (PMC9965764; doi:10.3390/plants12040881)
Supplement: Supplementary file 1 [file plants-12-00881-s001.zip › plants-2149076-supplementary.pdf]

Supplementary Materials

Table S1. 71 chemophenetic marker molecules selected by PLS-DA characterizing the investigated *Riccia* species. Columns include the internal identifier used by the XCMS peak detection software, the mass-to-charge ratio of the precursor ion (m/z), retention time (RT) (s), compound name, the most specific compound class, the SMILES, and the level of annotation confidence (MSI level) according to [37].

XCMS name	Precursor Mass (Da)	Retention Time (s)	Compound Name	Compound Class	SMILES	MSI level
FT0009_neg	44,998	920,251	-	-	-	-
FT0016_neg	59,014	304,491	-	-	-	-
FT0056_neg	112,986	514,019	-	-	-	-
FT0059_neg	113,025	420,632	-	-	-	-
FT0066_neg	116,929	305,891	-	-	-	-
FT0067_neg	116,929	279,664	-	-	-	-
FT0177_neg	165,019	343,250	-	-	-	-
FT0191_neg	173,009	210,976	-	-	-	-
FT0205_neg	175,025	374,208	-	-	-	-
FT0340_neg	219,103	386,633	-	-	-	-
FT0478_neg	257,073	301,674	-	Peptides	-	3
FT0483_neg	257,894	94,183	-	Polyhalopyridines	-	3
FT0539_neg	271,061	343,250	-	Indoles and derivatives	-	3
FT0624_neg	285,031	514,019	-	-	-	-
FT0705_neg	297,040	514,019	-	O-methylated flavonoids	-	3
FT0709_neg	297,124	302,779	-	-	-	-
FT0719_neg	299,052	514,019	-	-	-	-
FT0720_neg	299,056	478,661	Dinatin	O-methylated flavonoids	COC1=C(C2=C(C=C1O)OC(=CC2=	3
FT0721_neg	299,056	374,273	-	-	-	-
FT0723_neg	299,150	258,724	-	-	-	-
FT0737_pos	219,211	786,065	-	Prenol lipids	-	3
FT0765_neg	303,160	281,317	-	Unsaturated fatty acids	-	3
FT0810_neg	313,072	420,650	-	-	-	-
FT0832_neg	315,077	514,019	-	Dipeptides	-	3
FT0833_neg	315,077	420,632	-	Dipeptides	-	3
FT0834_neg	315,087	300,338	-	Aryl chlorides	-	3
FT0839_neg	315,098	238,775	-	Phenol ethers	-	3
FT0902_neg	323,186	470,279	-	Methyl-branched fatty	-	3
FT0911_neg	325,202	464,049	-	-	-	-
FT0991_neg	335,186	565,247	-	Amino acids and	-	3
FT1113_neg	355,088	246,652	-	Benzene and substituted	-	3
FT1116_neg	355,088	46,998	-	Benzene and substituted	-	3
FT1330_pos	273,076	270,530	Thunberginol G	Indanes	C1C(OC(=O)C2=C1C=CC=C2O)C3	2
FT1331_pos	273,066	440,548	-	1-hydroxy-2-	-	3
FT1333_pos	273,076	249,296	-	1-hydroxy-2-	-	3
FT1337_pos	273,076	303,271	4,8-Dihydroxy-3-(4-	Indanes	C1=CC2=C(C(=C1)O)C(=O)OC(C2	2
FT1463_neg	403,115	333,042	-	-	-	-
FT1492_neg	409,183	369,567	-	Amino acids and	-	3
FT1610_neg	425,210	412,939	-	-	-	-
FT1633_neg	428,070	409,931	-	-	-	-
FT1670_neg	435,129	313,025	-	Hexoses	-	3
FT1723_pos	300,061	372,497	-	Pentoses	-	3
FT1725_pos	300,059	440,800	-	Pentoses	-	3
FT1726_pos	300,062	514,188	-	Pentoses	-	3
FT1765_pos	303,086	319,526	-	Naphthopyranones	-	3

FT1849_neg	462,099	188,035	8-[[2-(3,4-	Benzenesulfonamides	CC1=C(N=C(O1)C2=CC(=C(C=C2)	2
FT1877_neg	465,104	352,245	-	-	-	-
FT2010_neg	489,104	421,157	Pectolinarigenin 7-	Flavonoid-7-O-	COC1=CC=C(C=C1)C2=CC(=O)C3	2
FT2026_neg	491,109	420,650	-	N-acyl-alpha amino acids	-	3
FT2126_neg	509,070	409,931	-	-	-	-
FT2201_neg	521,130	258,737	-	-	-	-
FT2210_pos	333,204	582,982	-	Amino acids and	-	3
FT2267_neg	535,045	369,196	-	Aryl ketones	-	3
FT2269_neg	535,125	464,032	-	Glycosyl compounds	-	3
FT2397_neg	558,044	283,986	-	-	-	-
FT2409_pos	345,167	537,094	-	Alpha amino acids	-	3
FT2410_pos	345,167	539,776	-	Amino acids and	-	3
FT2427_neg	562,041	276,273	-	Diphenylethers	-	3
FT2498_neg	577,072	426,196	-	-	-	-
FT2867_pos	380,182	163,014	L-threonyl-L-prolyl-L-	Oligopeptides	CC(C(C(=O)N1CCCC1C(=O)NC(C	2
FT3032_neg	681,226	188,099	-	N-acyl-alpha amino acids	-	3
FT3311_neg	775,215	685,094	-	-	-	-
FT3617_neg	1071,289	76,277	-	-	-	-
FT3633_neg	1113,300	194,636	-	-	-	-
FT3719_pos	487,100	306,172	-	Phenolic glycosides	-	3
FT4852_pos	623,124	303,292	-	Peptides	-	3
FT4854_pos	623,116	435,697	-	Alpha amino acids and	-	3
FT5131_pos	649,443	942,538	-	Carbamate esters	-	3
FT5147_pos	650,447	942,538	-	Hydroxysteroids	-	3
FT5242_pos	661,414	811,314	-	Sesquiterpenoids	-	3
FT5243_pos	661,405	822,266	-	Alpha amino acids and	-	3

Table S2. Compounds of interest. Table containing known compounds that were previously described in literature to be characteristic for *Riccia* species.

XCMS name	Compound name	Precursor Mass (Da)	Polarity	RT (s)	CSI:FingerID Score	SMILES
FT0782_neg	Eriodictiol	287,06	negative	279,6	-50,24	C1C(OC2=CC(=CC(=C2C1=O)O)O)C3=CC(=C(C=C3)O)O
FT0782_neg	Steppogenin	287,06	negative	279,6	-82,522	C1C(OC2=CC(=CC(=C2C1=O)O)O)C3=C(C=C(C=C3)O)O
FT0789_neg	Eriodictiol	287,06	negative	240,6	-50,24	C1C(OC2=CC(=CC(=C2C1=O)O)O)C3=CC(=C(C=C3)O)O
FT0789_neg	Steppogenin	287,06	negative	240,6	-82,522	C1C(OC2=CC(=CC(=C2C1=O)O)O)C3=C(C=C(C=C3)O)O
FT2108_neg	Naringenin-7-O-glucuronide	447,09	negative	362,4	-120,299	C1C(OC2=CC(=CC(=C2C1=O)O)OC3C(C(C(C(O3)C(=O)O)O)O)O)C4=CC=C(C=C4)O
FT2236_neg	Luteolin 7-o-glucuronide	461,07	negative	332,4	-80,933	C1=CC(=C(C=C1C2=CC(=O)C3=C(C=C(C=C3O2)OC4C(C(C(C(O4)C(=O)O)O)O)O)O)O)O
FT2239_neg	Luteolin 7-o-glucuronide	461,07	negative	279,6	-80,933	C1=CC(=C(C=C1C2=CC(=O)C3=C(C=C(C=C3O2)OC4C(C(C(C(O4)C(=O)O)O)O)O)O)O)O
FT2240_neg	Luteolin 7-o-glucuronide	461,07	negative	349,2	-80,933	C1=CC(=C(C=C1C2=CC(=O)C3=C(C=C(C=C3O2)OC4C(C(C(C(O4)C(=O)O)O)O)O)O)O)O

Table S3. Chemophenetic biomarker molecules selected by PLS-DA representative of each of the investigated *Riccia* species. Columns include the internal identifier used by the XCMS peak detection software, the mass-to-charge ratio of the precursor ion (m/z), retention time (RT) (s), compound name, the most specific compound class, the SMILES, and the level of annotation confidence (MSI level) according to [37].

XCMS name	Precursor mass (Da)	Retention time (s)	Compound Name	Compound Class	SMILES	MSI level
FT0021_neg	61,988	626,524	-	-	-	-
FT0023_neg	61,988	582,661	-	-	-	-
FT0024_neg	61,988	279,884	-	-	-	-

FT0068_neg	112,986	626,524	-	-	-	-
FT0071_neg	112,986	600,723	-	-	-	-
FT0072_neg	112,986	582,661	-	-	-	-
FT0087_neg	116,928	499,702	-	-	-	-
FT0088_neg	116,929	582,593	-	-	-	-
FT0089_neg	116,929	626,524	-	-	-	-
FT0245_neg	175,025	216,383	-	-	-	-
FT0388_neg	219,102	578,388	-	-	-	-
FT0430_neg	231,066	356,802	6-acetyl-7-hydroxy-2,3-	Benzopyrans	c12c(cc(c(c2)C(=O)C)O)oc(c(	2
FT0483_neg	243,899	93,316	-	-	-	-
FT0547_neg	257,065	28,626	-	Alpha amino acids	-	3
FT0685_neg	281,176	495,228	-	Prostaglandins and related	-	3
FT0686_neg	281,176	526,396	-	Prostaglandins and related	-	3
FT0713_neg	285,040	342,071	Campherol	7-hydroxyflavonoids	C1=CC(=CC=C1C2=C(C(=O)	2
FT0814_neg	299,077	216,359	-	Phenolic glycosides	-	3
FT0834_neg	301,082	216,359	-	Boronic acid derivatives	-	3
FT0864_neg	303,158	526,423	-	Unsaturated fatty acids	-	3
FT0879_neg	305,176	533,415	-	Dicarboxylic acids and	-	3
FT0893_neg	307,191	477,886	-	Lineolic acids and	-	3
FT1016_neg	323,186	533,415	-	Methyl-branched fatty acids	-	3
FT1038_pos	237,091	557,967	-	Amino acids	-	3
FT1039_pos	237,095	71,863	-	Alpha amino acids and	-	3
FT1136_neg	337,809	212,443	-	Arylsulfonic acids and	-	3
FT1174_neg	343,067	216,359	Vanillic acidglucuronide	Hydrolyzable tannins	COC1=C(C=CC(=C1)C(=O)O	2
FT1284_neg	357,083	233,912	-	Aryl ketones	-	3
FT1573_pos	273,083	36,380	-	Benzoic acids and	-	3
FT1589_neg	398,014	51,885	-	Dichlorobenzenes	-	3
FT1756_neg	423,197	651,511	-	Gamma-glutamyl peptides	-	3
FT1812_neg	427,182	626,524	-	Iridoid O-glycosides	-	3
FT1819_neg	427,998	216,383	-	Purine nucleotides	-	3
FT1864_neg	435,123	499,651	-	Amino acids and derivatives	-	3
FT1870_neg	436,127	499,651	-	2-halobenzoic acids and	-	3
FT1875_neg	437,139	600,723	-	2-arylbenzofuran flavonoids	-	3
FT1891_pos	291,196	477,366	-	Lineolic acids and	-	3
FT1897_neg	439,154	499,727	-	O-glycosyl compounds	-	3
FT1899_neg	439,154	529,260	-	O-glycosyl compounds	-	3
FT1900_neg	439,155	585,081	-	O-glycosyl compounds	-	3
FT1901_neg	439,155	577,409	-	O-glycosyl compounds	-	3
FT1919_neg	441,161	529,337	-	Phenol ethers	-	3
FT1945_neg	443,177	582,593	-	Alpha amino acids and	-	3
FT1956_neg	444,994	216,359	-	Benzenesulfonic acids and	-	3
FT1992_neg	449,108	404,035	-	Benzodioxoles	-	3
FT2022_neg	453,170	642,024	-	Amino acids and derivatives	-	3
FT2030_pos	300,066	477,366	-	Cysteine and derivatives	-	3
FT2035_neg	454,174	642,024	-	3-oxosteroids	-	3
FT2049_neg	455,149	462,747	-	Alpha amino acids and	-	3
FT2050_neg	455,176	642,024	-	Amino acids and derivatives	-	3
FT2093_neg	461,072	342,026	Luteolin 7-glucuronide	Flavonoid-7-O-glucuronides	C1=CC(=C(C=C1C2=CC(=O)	2
FT2097_neg	461,136	529,337	-	Amino acids and derivatives	-	3
FT2099_neg	461,152	626,524	-	Alpha amino acids and	-	3
FT2118_neg	463,152	582,169	-	Alpha amino acid amides	-	3
FT2119_neg	463,152	585,139	-	Alpha amino acid amides	-	3
FT2167_neg	469,165	562,806	-	Amino acids and derivatives	-	3
FT2182_neg	471,173	499,702	-	O-glycosyl compounds	-	3
FT2236_neg	479,147	499,727	-	Alloxazines and	-	3

FT2238_neg	479,145	582,890	-	Alloxazines and	-	3
FT2296_neg	487,176	577,400	-	Alpha amino acids and	-	3
FT2321_neg	491,146	499,727	-	Glucuronides	-	3
FT2341_neg	493,163	626,524	-	Phenol ethers	-	3
FT2363_neg	496,145	172,882	-	Alpha amino acids and	-	3
FT2431_neg	505,126	532,878	-	Methoxybenzoic acids and	-	3
FT2460_neg	511,080	499,727	-	Phenol ethers	-	3
FT2482_neg	515,081	499,727	-	Amino acids and derivatives	-	3
FT2488_neg	515,145	626,524	-	Terpene glycosides	-	3
FT2512_neg	517,122	499,727	-	Alpha amino acids and	-	3
FT2514_neg	517,151	626,524	-	Phenol ethers	-	3
FT2579_neg	528,042	528,871	-	Alkylglucosinolates	-	3
FT2584_pos	333,203	539,926	-	Amino acids and derivatives	-	3
FT2591_pos	333,203	558,853	-	Amino acids and derivatives	-	3
FT2796_neg	559,210	614,055	-	Triterpenoids	-	3
FT2818_neg	562,004	536,291	-	Purine ribonucleoside	-	3
FT2853_neg	569,097	381,908	-	Anthracenecarboxylic acids	-	3
FT2862_neg	572,032	536,291	-	Amino acids and derivatives	-	3
FT3062_neg	606,059	529,260	-	Amino acids and derivatives	-	3
FT3072_neg	607,166	335,946	-	Isoflavonoid O-glycosides	-	3
FT3073_neg	607,130	356,802	-	Flavonoid-3-O-glycosides	-	3
FT3088_neg	609,172	335,946	-	1-acyl-sn-glycerol-3-	-	3
FT3148_neg	621,145	345,997	-	Flavonoid O-glycosides	-	3
FT3266_neg	637,104	342,026	Luteolin 7,3''-diglucuronide	Flavonoid-7-O-glucuronides	C1=CC(=C(C=C1C2=CC(=O)	2
FT3348_neg	651,111	279,884	-	Alpha amino acids and	-	3
FT3350_neg	651,119	626,648	Luteolin 3'-methyl ether 7-	Flavonoid-7-O-glucuronides	COC1=C(C=CC(=C1)C2=CC(	2
FT3351_neg	651,119	356,846	Luteolin 3'-methyl ether 7-	Flavonoid-7-O-glucuronides	COC1=C(C=CC(=C1)C2=CC(	2
FT3363_neg	653,116	279,884	-	Flavonoid-3-O-glycosides	-	3
FT3389_neg	659,086	342,026	-	Flavonoid-7-O-glucuronides	-	3
FT3448_neg	669,249	626,992	-	Amino acids and derivatives	-	3
FT3543_neg	687,141	216,359	-	Purine nucleotide sugars	-	3
FT3718_neg	733,235	763,383	-	Phenolic glycosides	-	3
FT3738_neg	737,029	342,026	-	(5'->5')-dinucleotides	-	3
FT3754_neg	741,149	85,055	-	Flavonoid-3-O-glycosides	-	3
FT3765_neg	746,238	457,864	-	Gamma-glutamyl peptides	-	3
FT3967_neg	853,365	626,524	-	Amino acids and derivatives	-	3
FT4011_neg	880,203	279,884	-	Oligopeptides	-	3
FT4014_neg	881,332	582,776	-	Alkyl glycosides	-	3
FT4047_neg	901,297	529,471	-	Peptides	-	3
FT6093_pos	643,384	550,240	-	Peptides	-	3
FT6255_pos	659,378	745,548	-	Hydroxysteroids	-	3

Table S4. List of samples and their identification codes for use with the different types of analyses.

Species name	Taxon ID	Voucher ID	Collection date	Geographical coordinates	Text on voucher sleeve	Metabolomics sample ID positive mode	Metabolomics sample ID negative mode	Sequencing sample ID
Riccia glauca L.	*NCBI:129944, GBIF:5286298*	JE04010991	2021-09-13	48.638275 N, 9.2534083 E	Aichtal, Grötzingen, Acker,	R-glauca-1-<br>R-glauca-2-<br>R-glauca-3-	R-glauca-1-<br>R-glauca-2-<br>R-glauca-3-	Ri03
Riccia sorocarpa	*NCBI:122646, GBIF:5286296*	JE04010990	2021-09-13	48.638275 N, 9.2534083 E	Aichtal, Grötzingen, Acker,	R-sorocarpa-1-<br>R-sorocarpa-2-<br>R-sorocarpa-3-	R-sorocarpa-1-<br>R-sorocarpa-2-<br>R-sorocarpa-3-	Ri02

Riccia warnstorfii	*GBIF:5931123*	JE04010989	2021-09-13	48.638275 N, 9.2534083 E	Aichtal, Grötzingen, Acker,	R-wanstorfii-2- R-wanstorfii-3- R-wanstorfii-4-	R-wanstorfii-2- R-wanstorfii-3- R-wanstorfii-4-	Ri01
Lunularia cruciata (L.)	*NCBI:56931, GBIF:5286308*	JE04010993	2021-12-08	51.494848 N, 11.942323 E	IPB-Gelände, hinter Haus Heise	Lunularia-cruciata-1- Lunularia-cruciata-2- Lunularia-cruciata-3-	Lunularia-cruciata-1- Lunularia-cruciata-2- Lunularia-cruciata-3-	Ri05

Reference

37. Peters, K.; Blatt-Janmaat, K.; Tkach, N.; Van Dam, N.M.; Neumann, S. Investigating untargeted metabolomics for its use in integrative taxonomy—Linking metabolomics, DNA marker-based se-quencing and bioimaging of phenotypes. *Zenodo* **2023**. https://doi.org/10.5281/ZENODO.7638304.
